# Early Neurological Deterioration in Subcortical Infarcts: A Narrative Review

**DOI:** 10.3390/brainsci16050437

**Published:** 2026-04-22

**Authors:** Juan José Mengual, Carmen Montalvo, Sandra Boned, Carla Avellaneda-Gómez, Manuel Gómez-Choco

**Affiliations:** Neurology Department, Complex Hospitalari Universitari Moisès Broggi, Institut d’Investigació Biomèdica de Bellvitge (IDIBELL), Oriol Martorell 12, 08970 Sant Joan Despí, Spain; jjmengualc@csi.cat (J.J.M.); cmontalvoo@csi.cat (C.M.); sbonedr@csi.cat (S.B.); cavellanedag@csi.cat (C.A.-G.)

**Keywords:** subcortical infarct, early neurological deterioration, lacunar infarct, branch atheromatous disease, small vessel disease

## Abstract

**Highlights:**

**What are the main findings?**

• Early neurological deterioration affects ~20–25% of patients with a single small subcortical infarction and worsens functional outcomes.
• Hemodynamic impairment, particularly related to proximal perforator pathology and perfusion deficits, is a key mechanism underlying END.

**What are the implications of the main findings?**

• END should be considered a dynamic and potentially treatable process rather than an inevitable progression.
• Early identification of high-risk patients may enable targeted preventive and rescue strategies, although high-quality randomized evidence is still needed.

**Abstract:**

**Background/Objectives:** Early neurological deterioration (END) is a frequent and clinically relevant complication in patients with a single small subcortical infarction (SSI), including lacunar infarction and branch atheromatous disease (BAD). Despite initially mild symptoms, END occurs in approximately 20–25% of cases and is strongly associated with poor functional outcomes. However, definitions, mechanisms, predictors, and therapeutic strategies remain heterogeneous. This review aims to synthesize current evidence regarding the incidence, pathophysiology, predictors, and management of END in SSI. **Methods:** We performed a narrative review of published studies addressing END in patients with lacunar stroke or SSI. We analyzed data on END definitions and incidence, imaging and clinical predictors, proposed pathophysiological mechanisms, and preventive and rescue therapeutic strategies. **Results:** END definitions vary across studies, most commonly defined as a ≥2-point increase in the National Institutes of Health Stroke Scale within 48–72 h. Hemodynamic compromise due to proximal perforator pathology, particularly in BAD, appears central to END development. Advanced imaging studies demonstrate perfusion abnormalities beyond the infarct core, supporting the concept of a “lacunar penumbra.” Lesion topology, proximal infarct patterns, parent artery plaques, larger infarct size, and vertical extension are consistent imaging predictors. Clinical factors such as diabetes mellitus, higher baseline severity, systemic inflammation, and increased arterial stiffness further modulate risk. Preventive strategies, including early dual antiplatelet therapy and intensified antithrombotic regimens, show promising signals, while induced hypertension may benefit selected patients as a rescue therapy. However, evidence remains largely observational or derived from subgroup analyses. **Conclusions:** END in SSI is a multifactorial and potentially modifiable process driven by interactions between proximal vascular pathology, hemodynamic failure, and tissue vulnerability. Standardized definitions, MRI-based phenotyping, and mechanism-driven trials are needed to optimize risk stratification and develop targeted preventive and rescue strategies.

## 1. Introduction

Around 25% of ischemic strokes are caused by small vessel pathology. Owing to the limited volume of brain tissue involved, these strokes are generally associated with a more favorable prognosis than ischemic strokes related to cardioembolic or large artery disease [1]. In 2013, the term recent small subcortical (RSSI) infarct was first introduced as part of the STRIVE criteria [2] to describe acute subcortical infarcts located in the territory of deep perforating arteries arising from the circle of Willis, with a maximal axial diameter of ≤20 mm. The etiology of RSSI is heterogeneous, but most cases are attributed to either cerebral small vessel disease (cSVD) or branch atheromatous disease (BAD). In contrast to cSVD, which is thought to reflect intrinsic pathology of distal perforating arterioles, such as lipohyalinosis and fibrinoid necrosis, BAD is caused by atherosclerotic plaques involving the origin of a perforating artery or the parent artery from which it arises [3]. Given this etiological overlap and the limitations of conventional stroke classification systems in reliably distinguishing between cSVD and BAD, several studies have adopted the broader term single subcortical infarct (SSI) to describe an isolated ischemic lesion affecting the territory of deep perforating arteries arising from the circle of Willis [4].

## 2. Methods

This narrative review was conducted to summarize current evidence regarding early neurological deterioration (END) in patients with SSI, including lacunar infarction and branch atheromatous disease (BAD). A structured, non-systematic literature search was performed in PubMed/MEDLINE up to January 2026 using combinations of the following keywords: “lacunar stroke”, “single small subcortical infarction”, “branch atheromatous disease”, “early neurological deterioration”, “progressive lacunar stroke”, “capsular warning syndrome”, “perfusion”, “parent artery plaque”, “dual antiplatelet therapy”, “argatroban”, “tirofiban”, and “induced hypertension”. Reference lists of relevant articles were manually screened to identify additional studies. Studies were selected based on their relevance to the topic. Given the narrative nature of this review, no formal quality assessment or predefined inclusion criteria were applied.

We included observational studies, randomized clinical trials, and mechanistic neuroimaging studies that specifically evaluated END incidence, predictors, pathophysiological mechanisms, or therapeutic strategies in patients with lacunar stroke or SSI. Studies focused exclusively on large vessel occlusion or cardioembolic stroke were excluded unless subgroup analyses of small vessel disease were reported. Given the heterogeneity in END definitions and study designs, data were synthesized qualitatively. Particular emphasis was placed on studies with MRI-confirmed SSI and clearly defined END criteria. This review does not represent a formal systematic review or meta-analysis.

## 3. Early Neurological Deterioration

END is generally defined as clinical worsening attributable to ischemic progression in patients with acute ischemic stroke. In patients with lacunar stroke, the reported prevalence of END varies widely, but most studies consistently report rates of approximately 20–25% [4,5,6,7,8,9,10,11,12,13]. Clinically, END may present either as a progressive and sustained neurological worsening or as fluctuating symptoms, classically described as capsular warning syndrome. In line with the European Stroke Organisation guideline on lacunar ischemic stroke, these clinical patterns are often grouped under the broader concept of progressive lacunar stroke, encompassing END, progressive or stuttering stroke, and capsular warning syndrome [14]. Importantly, progressive neurological worsening should be distinguished from early recurrent stroke, as the underlying mechanisms and therapeutic implications may differ.

The occurrence of END is consistently associated with worse functional outcomes at 3 months [4]. Despite its clinical relevance, however, the definition of END remains heterogeneous across studies, varying both in the magnitude of neurological worsening and in the time window considered. A deterioration of ≥2 points on the National Institutes of Health Stroke Scale (NIHSS) is the most commonly used threshold [4,7,8,9,15,16,17,18,19] and has been shown to provide the lowest between-study heterogeneity, although some studies have adopted cutoffs of ≥1 or ≥4 points [12,20,21,22,23]. Similarly, while END is most often assessed within the first 48–72 h after stroke onset [5,6,8,11,15,18], some studies extend the observation period up to 7 days [12,16,17,19,20,21,22,24,25,26]. Table 1 shows the different END definitions and incidence across different studies.

The substantial heterogeneity in END definitions across studies has important methodological and clinical implications. Variability in NIHSS thresholds and time windows can significantly influence reported incidence rates, with lower thresholds and longer observation periods generally yielding higher END frequencies. This lack of standardization complicates comparisons across studies and may partially explain discrepancies in reported associations and treatment effects. In addition, heterogeneity in END definitions poses a major challenge for the design and interpretation of clinical trials, as differences in outcome definitions can affect event rates, statistical power, and the apparent efficacy of therapeutic interventions. Similarly, meta-analyses pooling studies with divergent END criteria may yield biased or difficult-to-interpret results. These limitations highlight the need for consensus definitions to improve comparability and facilitate the development of mechanism-based therapeutic strategies.

## 4. Pathophysiological Mechanisms and Contributing Factors of Early Neurological Deterioration

Hemodynamic compromise, defined as a reduction in effective cerebral blood flow in the territory supplied by affected perforating arteries due to impaired antegrade flow and limited collateral compensation, is considered one of the main drivers of END in patients with SSI. Perforating arteries are functional end-arteries with minimal or no collateral supply, rendering the tissue they supply particularly vulnerable to fluctuations in cerebral perfusion pressure. In the setting of BAD, an atherosclerotic plaque located at the origin of the perforating branch may result in critical flow limitation, especially during the acute phase, when cerebral autoregulatory mechanisms may be impaired.

**Figure 1:**
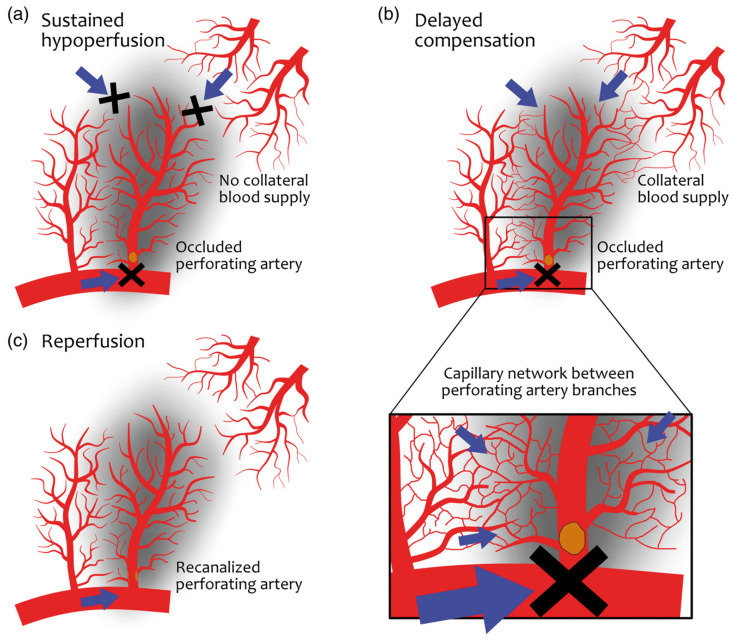
Hypothetical perfusion mechanisms in recent small subcortical infarcts (RSSIs). Reprinted with permission from Ref. [31]. Copyright 2018 Sage Publications. Schematic representation of proposed perfusion patterns in RSSIs supplied by a perforating artery (e.g., lenticulostriate artery) arising from a parent large artery (e.g., middle cerebral artery). A parallel perforator and adjacent vascular territories (e.g., medullary arteries) are illustrated to depict potential collateral pathways: (**a**) Sustained hypoperfusion: permanent occlusion of the perforating artery without effective collateral supply, resulting in a persistent reduction in regional perfusion within the infarct territory. (**b**) Delayed collateral compensation: absence of antegrade flow through the occluded perforator, but presence of microvascular anastomoses between neighboring perforating branches and adjacent vascular territories, allowing partial and delayed perfusion through capillary networks. (**c**) Reperfusion pattern: restoration of antegrade blood flow following spontaneous recanalization of an occluding thrombus, leading to re-establishment of perfusion within the affected perforator territory. These perfusion phenotypes illustrate potential hemodynamic substrates underlying different clinical trajectories and may contribute to the development of early neurological deterioration.

Supporting this concept, Yamada et al. reported that in patients with lacunar infarction in the lenticulostriate artery territory, the presence of perfusion deficits was significantly associated with subsequent neurological deterioration [28]. Subsequent perfusion imaging studies using CT perfusion and MRI-based techniques have confirmed focal hypoperfusion in a substantial subset of patients with acute lacunar infarction, with reported rates of approximately 40–60% in patients with confirmed SSI [29,30]. Despite the small volume of hypoperfusion, Rudilosso et al. described the presence of perfusion mismatch in approximately 42% of patients with lacunar stroke. Interestingly, clinical deterioration was more frequent among patients without a perfusion mismatch, highlighting the heterogeneity of the mechanisms underlying END in this population [29]. In a subsequent CT perfusion study including 67 patients with SSI, the same authors identified three distinct perfusion patterns (Figure 1): sustained hypoperfusion (48%), delayed compensation (16%), and reperfusion (27%). Sustained hypoperfusion (Figure 1a) likely reflects persistent occlusion of the perforating artery in the absence of effective collateral supply, whereas delayed compensation (Figure 1b) may indicate collateral flow from adjacent arterioles. The reperfusion pattern (Figure 1c), in turn, suggests spontaneous recanalization of a previously occluded vessel accompanied by distal vasodilation. Although sustained hypoperfusion was associated with higher systolic blood pressure values, none of these patterns was independently associated with END or clinical outcome, possibly reflecting limited statistical power [31]. Figure 1 shows the different suggested perfusion patterns in SSI.

Similar perfusion phenotypes have been replicated using perfusion MRI. In a study including 103 patients with SSI, three profiles were identified: normal perfusion (24%), compensated perfusion (30%), and hypoperfusion (46%). Patients with hypoperfusion or compensated perfusion patterns experienced higher rates of END compared with those with normal perfusion, whereas functional outcomes at 3 months were similar across groups [11]. Notably, hypoperfusion and compensated perfusion patterns were more frequently observed in patients with BAD and were not associated with the burden of MRI markers of cSVD, supporting the concept that hemodynamic impairment in this setting is driven by proximal perforator involvement rather than diffuse arteriolar pathology. In line with this hypothesis, Vynckier et al. demonstrated that the presence of hypoperfusion on perfusion imaging was independently associated with an increased risk of END in patients with lacunar stroke [9].

Other mechanisms, including thrombus propagation, local edema, systemic inflammation, and blood–brain barrier disruption, have also been proposed as contributors to END in patients with single subcortical infarction; however, the supporting evidence remains limited and largely indirect [5,6,32].

Given the strong association between BAD and END, together with the more atherosclerotic substrate underlying BAD, progression of an intraluminal thrombus at the origin of the perforating artery has been hypothesized to play a role in SSI-related END. Indirect support for this hypothesis comes from observational studies reporting lower rates of END in patients treated with dual antiplatelet therapy (DAPT) compared with single antiplatelet therapy or even intravenous thrombolysis [33].

It has also been suggested that the burden of cSVD-related lesions may contribute to END by reducing cerebral compensatory capacity through impaired vasodilatory mechanisms or diminished functional reserve. In a study including 435 patients with SSI, greater leukoaraiosis severity was independently associated with the occurrence of END [27], and similar results were observed by Nannoni et al. [34]. Furthermore, in a large cohort of 4424 patients with acute ischemic stroke, a higher burden of cSVD markers was associated with a reduced capacity for early clinical improvement, defined as a decrease of ≥3 points on the NIHSS at 24 h and at discharge. This association was particularly pronounced in patients with lacunar stroke, suggesting that diffuse microvascular damage may limit recovery even when infarct size is small [35].

END has also been associated with systemic inflammatory markers, suggesting a potential role of inflammation in modulating early ischemic progression. Several studies have reported that elevated inflammatory biomarkers, including leukocyte count, C-reactive protein, and neutrophil-to-lymphocyte ratio, are associated with an increased risk of neurological deterioration in patients with lacunar stroke [6,8]. Beyond being a marker of stroke severity, systemic inflammation may contribute to END through endothelial dysfunction, impaired microvascular perfusion, and increased blood–brain barrier permeability [36], thereby amplifying tissue vulnerability in perforating artery territories. However, whether inflammation plays a direct causal role or represents an epiphenomenon of infarct progression remains uncertain. In a study including 46 patients with lacunar stroke, admission leukocyte count and fibrinogen levels were independently associated with the occurrence of END, supporting a potential link between systemic inflammation and early neurological worsening [6].

In addition, cytotoxic and vasogenic edema within compact white matter structures has been proposed as a potential contributor to END in patients with SSI. This mechanism may be particularly relevant in anatomically constrained regions such as the internal capsule or ventral pons, where densely packed motor pathways offer limited tolerance to even small volume changes. This hypothesis is supported by classical anatomical observations of disproportionate clinical deficits caused by small lesions in these regions, the frequent dissociation between clinical deterioration and infarct growth on imaging, and emerging evidence of blood–brain barrier dysfunction and inflammation-mediated tissue injury in lacunar stroke. Nevertheless, direct causal proof for this mechanism remains lacking [5].

Blood–brain barrier disruption has been consistently demonstrated in patients with lacunar stroke using dynamic contrast-enhanced MRI and has been associated with worse functional outcomes and a greater burden of cSVD [32]. Aging-related alterations in blood–brain barrier integrity, including increased permeability and impaired endothelial function, may further exacerbate tissue vulnerability and contribute to a higher risk of END in older patients [37].

**Figure 2:**
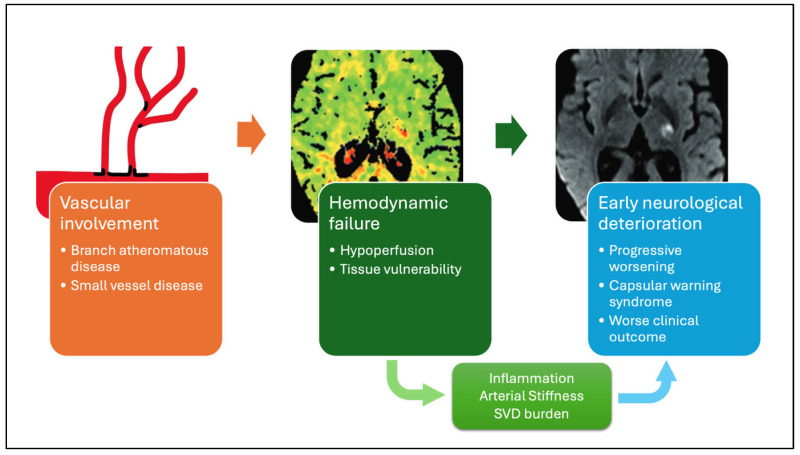
Mechanistic model of early neurological deterioration (END) in a single small subcortical infarction (SSI). Schematic representation of the multifactorial mechanisms contributing to END in patients with SSI. Proximal perforator pathology, particularly branch atheromatous disease, may cause critical flow limitation at the origin of deep perforating arteries, leading to hemodynamic compromise in territories with minimal collateral supply. Perfusion impairment may predispose to stepwise or progressive neurological worsening. Systemic and vascular modifiers, including arterial stiffness, small vessel disease burden, and systemic inflammation, interact with lesion-related factors to determine clinical evolution. Importantly, these pathophysiological mechanisms may have direct therapeutic implications. Different perfusion profiles likely reflect distinct biological substrates and may respond differently to targeted interventions. For instance, patients with sustained hypoperfusion may benefit from strategies aimed at improving cerebral perfusion, such as induced hypertension, whereas those with reperfusion patterns may be less likely to respond to hemodynamic modulation. Similarly, the presence of a “lacunar penumbra” suggests that early interventions targeting microvascular perfusion could potentially prevent progression in selected patients. In addition, blood–brain barrier dysfunction may represent a promising therapeutic target, as it could contribute to secondary injury through inflammatory and edematous mechanisms. Although current evidence remains limited, strategies aimed at stabilizing endothelial function and reducing permeability could have a role in mitigating early neurological deterioration.

Taken together, these data support a multifactorial model of END in SSI, in which proximal vascular pathology, hemodynamic failure, and tissue-level vulnerability interact to determine clinical evolution (Figure 2).

## 5. Predictors of END

Neuroimaging features are among the most consistent predictors of END. Beyond infarct size, several studies have shown that lesion topology and growth patterns are critical determinants for END [4,7,9,18,19]. Larger infarct area on diffusion-weighted imaging (DWI), greater vertical extension across consecutive slices, and lesion growth over time have all been independently associated with END [16,17,19]. Importantly, the pattern of lesion location relative to the parent artery appears to be particularly relevant. Infarcts extending to the basal surface of the parent artery, commonly referred to as proximal single small subcortical infarcts, are associated with a higher risk of END compared with distal lesions [18,38]. These proximal patterns are frequently accompanied by ipsilateral intracranial atherosclerosis and are thought to reflect BAD rather than pure distal small vessel pathology [10,18,39]. The presence of an atherosclerotic plaque at the origin of the perforating branch is the strongest predictor of END, independent of the degree of luminal stenosis, but no clear association has been found between the presence of neuroimaging cSVD markers and the development of END [4].

Beyond structural imaging, hemodynamic factors play a key role in END. Advanced imaging studies have demonstrated that some patients with SSI exhibit perfusion deficits extending beyond the DWI-defined infarct core, particularly when functionally eloquent regions such as the corticospinal tract are involved. These perfusion abnormalities have been strongly associated with subsequent neurological deterioration, even after adjustment for baseline stroke severity and infarct size [9,15,28,29]. These observations support the concept of a “lacunar penumbra”, in which hypoperfused yet potentially salvageable tissue contributes to stepwise or progressive clinical worsening. In this context, END may reflect a failure of compensatory microvascular perfusion rather than irreversible tissue damage at presentation.

The independence of these markers from clinical variables varies across studies. Some of these markers, particularly proximal infarct patterns and parent artery plaques, have shown independent associations with END after adjustment for baseline clinical severity and vascular risk factors, whereas others may partly reflect underlying stroke severity. In addition, external validation of these imaging predictors remains limited, as most evidence derives from single-center or relatively small cohort studies. Interestingly, conventional markers of cerebral small vessel disease, such as white matter hyperintensities and lacunes, have not been consistently associated with END, suggesting that acute lesion characteristics and local vascular pathology may play a more prominent role than chronic microvascular burden in determining early clinical progression.

Systemic vascular factors further modulate the risk of END. Among them, arterial stiffness, measured by brachial–ankle pulse wave velocity, has emerged as a strong independent predictor [16]. Elevated arterial stiffness is associated with an increased risk of END, larger infarcts, and greater vertical lesion extension. This finding provides a mechanistic link between END and endothelial dysfunction, impaired autoregulation, and blood–brain barrier disruption, which may render perforating artery territories particularly vulnerable to ischemic progression. In the same line, an increased pulsatility index measured with transcranial Doppler, which in part translates into increased arterial stiffness, is also associated with an increased risk of END [8].

Several clinical factors have been consistently associated with END. Higher baseline neurological severity, even within the low NIHSS range typical of SSI, increases the risk of END, likely reflecting a larger or more strategically located ischemic burden [4,18], although some other studies have found a higher risk of END in patients with low NIHSS [9]. Diabetes mellitus has repeatedly been identified as an independent predictor, potentially through its effects on endothelial function, microvascular integrity, and infarct expansion [5,24]. Female sex has also been reported as an independent predictor in large prospective cohorts, although the mechanisms underlying this association remain unclear [18]. In addition to clinical severity and vascular risk factors, systemic inflammatory markers have also been associated with END [6]. Specifically, a higher neutrophil-to-lymphocyte ratio was independently related to neurological deterioration [8].

In contrast, the role of age and acute blood pressure levels appears less consistent across studies and often loses significance after multivariable adjustment, suggesting that these factors may act as modulators rather than primary drivers of END [13]. A list of factors more consistently associated with END across the studies is shown in Table 2.

## 6. Prevention and Treatment of END

Therapeutic strategies for END can be broadly divided into preventive strategies aimed at reducing the risk of deterioration and rescue therapies intended to improve outcomes after END has occurred.

### 6.1. Preventive Strategies

Nowadays, DAPT has become the standard of care for many patients with non-cardioembolic ischemic stroke presenting with minor symptoms or who are not eligible for intravenous thrombolysis. In a prespecified post hoc analysis of the ARAMIS trial, a randomized study comparing DAPT with intravenous thrombolysis in patients with minor non-disabling ischemic stroke without large vessel occlusion, early DAPT significantly reduced the incidence of END compared with intravenous alteplase. Although ARAMIS was not designed specifically for small vessel disease, approximately one-quarter of the study population was classified as having small artery occlusion, and early DAPT significantly reduced the incidence of END in patients without large vessel occlusion [40,41]. This effect was observed to be particularly strong when DAPT was initiated within 3 h after symptom onset [42]. However, a substantial proportion of patients with SSI continue to develop END, and alternative strategies aimed at preventing END have been explored.

Cilostazol, a phosphodiesterase III inhibitor with antiplatelet, vasodilatory, and endothelial-modulating properties, has been investigated as a preventive strategy for END in the acute phase of ischemic stroke. Beyond platelet inhibition, cilostazol exerts pleiotropic effects on endothelial function, cerebral microcirculation, and neuroprotection, providing a biological rationale for its potential role in stabilizing small vessel ischemia. Evidence regarding its efficacy in preventing END, however, remains mixed and highly dependent on the population studied. In a randomized trial including 100 patients with perforating artery infarction treated within 48 h, cilostazol combined with ozagrel did not significantly reduce the incidence of END compared with ozagrel alone, despite a numerical reduction in END rates [43]. In contrast, a smaller randomized study of 76 patients with acute non-cardioembolic ischemic stroke reported that aspirin plus cilostazol significantly reduced the incidence of END or stroke recurrence compared with aspirin alone and was associated with improved functional outcomes at 6 months [44].

Although this study was not designed to specifically address SSI, around 47% were classified as small vessel disease according to the TOAST (Trial of Org 10172 in Acute Stroke Treatment) classification, a widely used etiological system that categorizes ischemic stroke subtypes based on clinical and imaging features [45]. Taken together, current data indicate that while cilostazol is safe and biologically plausible in the acute phase of ischemic stroke, robust evidence supporting its role in preventing END specifically in SSI is lacking. Its use in this context should therefore be considered investigational, pending confirmation in adequately powered, mechanism-oriented trials.

Emerging evidence supports a potential preventive role for argatroban, a direct thrombin inhibitor with antithrombotic and potential microcirculatory effects, as a preventive strategy in patients at high risk of END. When combined with DAPT in patients with BAD, in a nonrandomized observational study including 80 patients with BAD treated within 48 h of symptom onset, the combination of DAPT therapy plus argatroban was associated with a markedly lower incidence of END compared with DAPT alone (4.0% vs. 34.5%), as well as a substantially lower rate of poor functional outcome at 3 months (mRS ≥ 2: 4.0% vs. 38.2%). These associations remained significant after multivariable adjustment, and no increase in bleeding complications was observed in the combination therapy group [46]. These results will be tested in a future randomized clinical trial (ChiCTR2100046487).

Tirofiban, a potent glycoprotein IIb/IIIa inhibitor that blocks the final common pathway of platelet aggregation, has been proposed as a potential strategy to prevent END when administered early in patients with SSI. In patients with non-cardioembolic ischemic stroke, tirofiban has been shown to reduce the incidence of END compared with aspirin and to improve early clinical outcomes, supporting its potential role in stabilizing neurological progression in this population [47]. The BRANT trial (NCT06037889) is a randomized study that evaluated early tirofiban in patients with BAD-related stroke involving the lenticulostriate or paramedian pontine arteries. Although the trial has been completed, the results are not yet available. This trial enrolled patients who underwent brain MRI within 48 h of symptom onset and randomized them to receive tirofiban for 72 h followed by standard antiplatelet therapy or standard antiplatelet therapy alone. The standard antiplatelet regimen included either aspirin (150–300 mg daily) or DAPT with aspirin (100 mg) plus clopidogrel (75 mg) [21].

### 6.2. Rescue Therapies

Alternative strategies have been explored for patients who develop END despite optimal antithrombotic therapy. Among them, DAPT has also emerged as a potentially effective approach. In a cohort study including 458 patients with lacunar stroke who experienced clinical deterioration, DAPT was associated with greater improvement in NIHSS scores at discharge compared with other treatment strategies, including intravenous thrombolysis. Moreover, DAPT was linked to better functional outcomes assessed by the modified Rankin Scale at multiple time points and to a significant reduction in clinical fluctuations, supporting its role in mitigating END [33].

Pharmacologically induced hypertension (PIH) has been proposed as a rescue therapy for patients who develop END. In a large cohort study including 4818 patients with lacunar stroke, END occurred in 147 patients, of whom 79 were treated with intravenous phenylephrine to induce PIH. Phenylephrine was administered for 24 h and gradually tapered after neurological improvement or after a maximum of 5 days of treatment. Compared with argatroban therapy, patients treated with PIH showed higher rates of END recovery and better functional outcomes, without an increase in mortality or intracranial hemorrhage [48]. Consistent findings were reported in a nationwide, prospective, multicenter registry from South Korea, including 3067 patients with END due to noncardioembolic ischemic stroke. In this cohort, 60% of patients received conservative management, 24.4% underwent antithrombotic modification, and 15.7% were treated with induced hypertension. Patients receiving induced hypertension had higher rates of neurological improvement compared with those managed conservatively or with antithrombotic changes, and it has also demonstrated better functional outcomes at 3 months [49]. Although these findings suggest a potential benefit of PIH as a rescue strategy, evidence is derived from observational studies, and randomized trials are needed to define optimal patient selection, timing, and blood pressure targets. A summary of the main therapeutic strategies is shown in Table 3.

Overall, the level of evidence supporting current therapeutic strategies for END in SSI remains limited. Most available data are derived from observational studies or subgroup analyses of randomized trials not specifically designed for small subcortical infarction. Among preventive strategies, early DAPT has the highest level of supporting evidence, mainly derived from randomized clinical trials in patients with minor ischemic stroke. In contrast, other approaches, including cilostazol, argatroban, and tirofiban, are supported by moderate to low levels of evidence, largely based on small randomized studies or observational data. Similarly, rescue strategies such as induced hypertension rely predominantly on low-level evidence from observational studies. These limitations highlight the need for dedicated randomized clinical trials specifically targeting patients with SSI.

## 7. Conclusions

END represents a frequent and clinically meaningful complication of SSI, substantially contributing to poor functional outcomes despite initially mild deficits. Accumulating evidence indicates that END is not a uniform phenomenon but rather the result of interacting lesion-related, hemodynamic, vascular, and systemic factors, with consistent signals implicating infarct topology and growth, perfusion impairment beyond the infarct core, and parent artery pathology. Importantly, END appears to be a dynamic and potentially modifiable process.

Preventive strategies based on early antiplatelet intensification have shown promising signals in populations relevant to small vessel and perforator-related stroke, particularly when initiated during the hyperacute phase, while rescue approaches targeting cerebral perfusion, such as induced hypertension, have demonstrated benefit once END has occurred. However, the current evidence remains fragmented, largely indirect, and predominantly derived from observational studies or subgroup analyses of randomized trials not specifically designed for SSI populations. In addition, substantial heterogeneity in END definitions (e.g., NIHSS thresholds, motor versus global scales, and time windows) limits comparability across studies and may influence both reported incidence rates and treatment effects.

Several additional limitations should be acknowledged. First, most studies rely on conventional imaging approaches, with limited use of advanced MRI or perfusion techniques to better characterize underlying mechanisms such as hemodynamic compromise or blood–brain barrier dysfunction. Second, many proposed imaging and clinical predictors have not been consistently validated in external cohorts or adjusted for potential confounders, raising concerns about their generalizability and independent predictive value. Third, the distinction between lacunar infarction and branch atheromatous disease remains challenging in routine clinical practice, potentially leading to heterogeneous study populations. Finally, the lack of standardized therapeutic protocols and the variability in timing and selection of interventions further complicate the interpretation of available data.

Future studies should therefore prioritize the adoption of standardized and clinically meaningful definitions of END, ideally incorporating both global and domain-specific neurological changes. Advanced neuroimaging, including high-resolution vessel wall imaging and perfusion techniques, should be integrated to enable mechanism-based phenotyping of SSI. Prospective, adequately powered randomized clinical trials specifically targeting SSI populations are urgently needed to evaluate both preventive and rescue strategies, with stratification based on imaging and clinical risk profiles. In parallel, the incorporation of biomarkers related to inflammation, endothelial dysfunction, and blood–brain barrier integrity may further improve risk stratification and identify novel therapeutic targets.

Advancing this field will be essential to move beyond passive observation toward individualized, mechanism-driven, and proactive management of END in small vessel and perforator-related stroke.

## Figures and Tables

**Table 1:** Definitions and incidence of END across studies in patients with SSI. END definitions and incidence rates are reported as described in the original studies and vary according to the NIHSS thresholds, neurological domains assessed, and time windows after stroke onset. Abbreviations: END, early neurological deterioration; NIHSS, National Institutes of Health Stroke Scale; DWI, diffusion-weighted imaging; MCA, middle cerebral artery; LSA, lenticulostriate artery.

Study	Population	Definition of END	END Incidence
Definition: ≥2-point increase in total NIHSS score			
Terasawa et al., 2008 [19]	72 patients with acute supratentorial small vessel infarction	Increase of ≥2 points in total NIHSS score within 7 days after stroke onset	21%
Kim et al., 2011 [26]	168 patients with DWI-confirmed lacunar motor syndrome	Increase of ≥2 points in total NIHSS score within 5 days after stroke onset	28%
Takase et al., 2011 [17]	40 patients with lacunar infarction in the lenticulostriate artery territory	Increase of ≥2 points in the NIHSS motor score within 7 days after stroke onset	47.5%
Huang et al., 2014 [15]	43 patients with single small subcortical infarction	Increase of ≥2 points in total NIHSS score within 72 h after stroke onset	23.3%
Duan et al., 2015 [18]	22 patients with single small subcortical infarction in the MCA perforator territory	Increase of ≥2 points in total NIHSS score within 72 h after stroke onset	26.4%
Jeong et al., 2015 [4]	587 patients with single small subcortical infarction within 48 h	Increase of ≥2 points in total NIHSS score, or ≥1 point in level of consciousness, or ≥1 point in motor items within 3 weeks	13.5%
Li et al., 2019 [7]	407 patients with pontine infarction	Increase of ≥2 points in total NIHSS score or ≥1 point in motor items within 7 days	28%
Nam et al., 2021 [8]	438 patients with single subcortical infarction	Increase of ≥2 points in total NIHSS score or ≥1 point in motor items within 72 h	11.9%
Vynckier et al., 2021 [9]	365 MRI-confirmed lacunar infarctions within 12 h	Increase of ≥2 points in total NIHSS score within 24 h	16.7%
Yan et al., 2022 [10]	74 patients with single subcortical infarction in the LSA territory	Increase of ≥2 points in total NIHSS score or ≥1 point in motor items within 7 days	31.1%
Huang et al., 2022 [11]	103 patients with acute single small subcortical infarction	Increase of ≥2 points in total NIHSS score within 72 h	21%
Definition: ≥1-point increase in motor NIHSS items			
Nakamura et al., 1999 [23]	92 patients with acute supratentorial lacunar infarction	Increase of ≥1 point in summed NIHSS motor score (arm + leg) within 24 h	27%
Audebert et al., 2004 [6]	46 patients with clinical lacunar stroke admitted <12 h	Increase of ≥1 point in the NIHSS motor item within 72 h	23.9%
Kim et al., 2008 [25]	167 patients with acute deep subcortical infarction	Increase of ≥1 point in the NIHSS motor item within 7 days	13.8%
Nagakane et al., 2008 [22]	61 patients with DWI-confirmed lacunar infarction	Increase of ≥1 point in the NIHSS motor item within 7 days	26%
Yamamoto et al., 2010 [24]	60 patients with lacunar infarction in the LSA territory	Increase of ≥1 point in the NIHSS motor item within 7 days	43%
Composite or alternative definitions			
Serena et al., 2001 [5]	113 patients with acute lacunar infarction	Decrease of ≥1 point in motor items of Canadian Stroke Scale within 48 h	23.9%
Feng et al., 2014 [27]	204 patients with MRI-confirmed lacunar infarction	Increase of ≥1 point in total NIHSS score within 14 days	9.8%
Saji et al., 2012 [16]	156 patients with first-ever deep subcortical infarction	Increase of ≥2 points in total NIHSS score or ≥1 point in motor items within 7 days	33%
Li et al., 2024 [12]	476 patients with branch atheromatous disease	Increase of ≥4 points in total NIHSS score or ≥1 point in motor items within 7 days	14.7%

**Table 2:** Factors associated with END in patients with SSI. Factors are categorized according to their type and the strength of association (strong, moderate, or inconsistent), based on the consistency of findings across published studies. References shown are representative and not exhaustive. The classification is based on qualitative synthesis rather than pooled quantitative estimates. Abbreviations: DWI, diffusion-weighted imaging; BAD, branch atheromatous disease; NLR, neutrophil-to-lymphocyte ratio.

Factor	Type	Strength of Association
Larger infarct size (DWI) [16,17,19]	Radiological	Strong
Lesion growth over time [16,19]	Radiological	Strong
Greater vertical infarct extension [16,17,19]	Radiological	Strong
Proximal infarct pattern (BAD-like) [18,38]	Radiological	Strong
Parent artery pathology (plaque/stenosis) [4,10,39]	Radiological	Strong
Diabetes mellitus [5,24]	Clinical	Strong
Higher baseline neurological severity [4,18]	Clinical	Moderate
Perfusion deficits beyond DWI core [9,15,28,29]	Hemodynamic	Moderate
Ventral pontine infarction [7]	Radiological	Moderate
Fluctuating symptoms (capsular warning syndrome) [14]	Clinical	Moderate
Systemic inflammation (e.g., NLR) [6,8]	Systemic	Moderate
Increased arterial stiffness [16]	Systemic	Moderate
Female sex [4,18]	Clinical	Inconsistent

**Table 3:** Preventive and rescue therapeutic strategies for END in SSI. Abbreviations: AIS, acute ischemic stroke; BAD, branch atheromatous disease; DAPT, dual antiplatelet therapy; END, early neurological deterioration; LVO, large vessel occlusion; NIHSS, National Institutes of Health Stroke Scale; NS, not statistically significant; SVO, small vessel occlusion.

Study	Population	Strategy	Outcome Definition	Response	Study Design
END prevention					
Nakamura et al., J Neurol Sci 2012 [44]	76 patients with acute non-cardioembolic ischemic stroke (47% small-vessel disease)	Aspirin + cilostazol vs. aspirin	Increase of ≥1 point in NIHSS within 14 days	6% vs. 28%	Randomized pilot trial
Kondo et al., Eur Neurol 2013 [43]	100 patients with acute perforating artery infarction (lenticulostriate territory)	Cilostazol + ozagrel vs. ozagrel	Increase of ≥4 points in NIHSS within 7 days	12.5% vs. 16.0% (NS)	Randomized controlled trial
Xu et al., Stroke 2022 [46]	80 patients with branch atheromatous disease	DAPT + argatroban vs. DAPT	Increase of ≥2 points in NIHSS within 72 h	4.0% vs. 34.5%	Randomized controlled trial
Cui et al., Stroke 2024 [40]	Minor ischemic stroke without LVO	DAPT vs. intravenous thrombolysis	Increase of ≥4 points in NIHSS within 24 h	0.5% vs. 5.7%	Post hoc analysis of randomized controlled trial (ARAMIS)
Zhao et al., JAMA Neurol 2024 [47]	425 patients with non-cardioembolic AIS (29% SVO)	Tirofiban vs. aspirin	Increase of ≥4 points in NIHSS within 72 h	4.2% vs. 13.2%	Randomized controlled trial (TREND)
Rescue therapies					
Berberich et al., Stroke 2019 [33]	458 patients with lacunar stroke and established END	DAPT vs. standard antiplatelet therapy after END	Neurological improvement at discharge (NIHSS ≤ admission NIHSS)	68% vs. 36%	Observational cohort study
Park et al., BMC Neurol 2024 [48]	147 patients with acute lacunar stroke and END within 72 h (SVO + BAD)	Induced hypertension (phenylephrine) vs. argatroban	Decrease of ≥2 points in NIHSS within 7 days	77.2% vs. 51.5%	Prospective observational cohort
Kim et al., Stroke 2025 [49]	3067 patients with non-cardioembolic ischemic stroke and END due to ischemic progression (~20% SVO)	Induced hypertension vs. antithrombotic modification vs. conservative management	Decrease of ≥2 points in NIHSS before discharge	41.5% vs. 34.4% vs. 32.2%	Registry-based observational study

## Data Availability

No new data were created or analyzed in this study. Data sharing is not applicable to this article.
